# Investigating the dependence of the calibration parameter M on echo time

**DOI:** 10.1002/mrm.25603

**Published:** 2015-03-11

**Authors:** Hannah V. Hare, Daniel P. Bulte

**Affiliations:** ^1^ FMRIB CentreUniversity of OxfordOxfordUnited Kingdom

**Keywords:** calibration, BOLD, hypercapnia, echo time, MRI

## Abstract

**Purpose:**

The calculation of the calibration parameter M, which represents the maximum theoretically possible blood oxygen level dependent (BOLD) signal increase, is an essential intermediate step in any calibrated fMRI experiment. To better compare M values obtained across different studies, it is common to scale M values from their original BOLD echo time (TE) to a different echo time according to the theory that M is directly proportional to TE. To the best of our knowledge, this relationship has never been directly tested.

**Theory and Methods:**

A pseudocontinuous arterial spin labeling sequence with five readouts (TE ranging from 20 to 78 ms) was implemented to test the relationship between M and TE, both with and without the application of flow crushing gradients.

**Results:**

Both M and the BOLD signal were found to be linear functions of TE, but with a nonzero intercept. This intercept was reduced when crusher gradients were added, suggesting that the deviation from theory is a result of nonnegligible intravascular signal.

**Conclusion:**

The linear scaling method introduces some error when comparing M values acquired with different BOLD echo times. However, this error is small compared with other considerations, and would generally not preclude the continued use of this scaling method. Magn Reson Med 75:556–561, 2016. © 2015 The Authors. Magnetic Resonance in Medicine published by Wiley Periodicals, Inc. on behalf of International Society for Magnetic Resonance in Medicine.

## INTRODUCTION

Calibrated functional MRI 1 aims to look beyond the blood oxygen level dependent (BOLD) response and quantify the underlying physiological changes in cerebral metabolic rate of oxygen consumption (CMRO_2_). This is done by means of additional gas challenges such as hypercapnia 2 or hyperoxia 3, allowing the estimation of the calibration parameter M, which represents the maximum possible BOLD signal that could theoretically be evoked. From this it is possible to calculate the relative change in CMRO_2_ during a functional task from measured BOLD and cerebral blood flow (CBF) responses.

In an extension to the method described above, it has recently been shown that by presenting both hypercapnic and hyperoxic stimuli within a single session, it is possible not only to calculate relative changes in oxygen extraction fraction and CMRO_2_ but also to quantify their absolute, baseline values 4, 5, 6. This is potentially of great clinical interest, as it may allow for noninvasive, regional assessment of patients with a wide variety of pathologies.

The calculation of M as an intermediate calibration constant is key to all of the techniques described above. M is a physiological parameter that will vary depending on the type of tissue, field strength, voxel size, and physiology (including resting blood volume, hematocrit, and oxygen extraction fraction). Its value also depends on scanning parameters such as the echo time (TE), as this affects the size of the measured BOLD signal. It is commonly assumed that M is directly proportional to TE, allowing for experimental values to be linearly scaled for comparisons between studies 7. However, this has never been experimentally verified, and it is not known if other factors contributing to M may have some additional, nonlinear dependence on TE. This work aims to test the hypothesis that M varies linearly with TE.

## THEORY

The BOLD signal itself is sensitive to changes in the effective transverse relaxation rate, $R_{2}^{*}$, according to

(1) $$\frac{\Delta \text{BOLD}}{{\text{BOLD}}_{0}}=e^{-\text{TE}\cdot \Delta R_{2}^{*}}-1\approx -\text{TE}\cdot \Delta R_{}^{*}{}_{2}$$

This makes it particularly sensitive to the ratio of oxygenated (diamagnetic) to deoxygenated (paramagnetic) hemoglobin in the blood. It should be noted that Eq. [1] assumes negligible intravascular signal contribution, allowing the BOLD signal to be described by a simple, monoexponential expression, despite findings that intravascular spins contribute a significant fraction of observed BOLD signal changes [approximately 2/3 at 1.5T 8 and 1/3 at 3T 9].

Neurological stimuli result in localized increases in blood flow and/or increases in CMRO_2_, both of which affect $R_{2}^{*}$ and thus the measured BOLD signal. This relationship may be described by the following equation 10:

(2) $$\frac{\Delta \text{BOLD}}{{\text{BOLD}}_{0}}=M\left\{1-{\left(\frac{{\text{CMRO}}_{2}}{{\text{CMRO}}_{2}|{}_{0}}\right)}^{\beta }{\left(\frac{\text{CBF}}{{\text{CBF}}_{0}}\right)}^{\alpha -\beta }\right\}$$

where α is the Grubb coefficient relating changes in blood flow to blood volume, and β is a fitting parameter relating $R_{2}^{*}$ to vessel size. In the specific case of a hypercapnic stimulus, CMRO_2_ is assumed to remain unchanged. M is defined as A×TE×CBV_0_× ${\left[\text{dHb}\right]}_{\text{vo}}^{\beta }$, where the baseline cerebral blood volume CBV_0_, baseline venous deoxyhemoglobin concentration ${\left[\text{dHb}\right]}_{\text{vo}}$ and proportionality constant A are expected to be independent of echo time. Thus, M is predicted to be directly proportional to TE.

Equation [2] has been derived using several assumptions, including negligible deoxyhemoglobin concentration in arterial blood, a consistent flow‐volume coupling as first described by Grubb et al 11, and the semiempirical description of $R_{2}^{*}$ as a function of CBV and deoxyhemoglobin concentration 10, 12. It is also based on Eq. [1], which assumes no intravascular signal contribution, as mentioned above.

The aim of this work was to investigate whether experimentally acquired BOLD signal change and M vary in direct proportion with TE as predicted by the theory outlined above. In addition, the validity of the negligible intravascular signal assumption was explored by acquiring data with and without the use of flow crushing gradients to suppress signal from fast‐flowing spins.

## METHODS

A pseudocontinuous arterial spin labeling (ASL) sequence was implemented on a 3T Siemens Verio scanner with a 32‐channel head coil, with a multislice, multiecho readout. Six slices were acquired with five echoes per slice, at 20/35/49/64/78 ms respectively, with GRAPPA acceleration factor of 3, tag duration 1.4 s, postlabel delay 1.8 s and repetition time (TR) of 4 s. In‐plane resolution was 3.4 × 3.4 mm, with 5 mm slice thickness and a distance factor of 50%.

To investigate the effects of intravascular spins on the BOLD signal and thus on M, the sequence was designed with the option of adding bipolar flow crushing gradients immediately before the first readout. Where used, these were set to have a cutoff velocity of 1.9 cm/s. Where flow crushers were not used, they were replaced by a short, empty event block within the sequence; in this way, all echoes were acquired at the same echo times with crushers turned on and off. Higher SNR would have been achieved for the ASL with a TE shorter than 20 ms; however, this would not have allowed for the flow crushing gradients to be incorporated, and thus not enabled us to answer the key question on intravascular signal contribution.

Ethical approval was granted for this study by the Oxfordshire Research Ethics Committee, and eight healthy volunteers (five female; mean age 24 ± 2 years) were scanned after giving informed consent. The imaging protocol consisted of two 3‐min periods of hypercapnia (delivering a gas mixture of 10% CO_2_, 21% oxygen, and balance nitrogen) interleaved with three 2‐min periods of baseline (breathing medical air; 21% oxygen, balance nitrogen), resulting in a single scan time of 12 minutes. Both gas mixtures were delivered through a nasal cannula (dual Nare, Flexicare, Mountain Ash, UK) and thus mixed with room air, resulting in a CO_2_ stimulus of approximately 4%. This protocol was repeated twice, both with and without flow crushing gradients.

### Data Analysis

Postprocessing was performed using the FMRIB Software Library (FSL) and Matlab (The MathWorks Inc., Natick, MA). After motion correction 13 and brain extraction 14, highpass filtering was applied with a cutoff value of 10 s for ASL data (first echo only) and 300 s for BOLD (all five echoes). No spatial smoothing was applied. A general linear model was used to calculate voxelwise flow responses to hypercapnia from the first echo, and BOLD responses from all echoes. $R_{2}^{*}$ maps were created from time‐averaged signal at all 5 echo times for both crushed and noncrushed acquisitions. Monoexponential signal decay was assumed.

A gray matter region of interest (ROI) was created for each scan from voxels with significant tag‐control (resting) ASL signal *and* a significant BOLD response to hypercapnia in at least three of the five BOLD echoes, where “significant” was defined as the 40% of brain voxels with the highest z‐statistics for the relevant contrast. The first condition was used because good tag‐control contrast during ASL is indicative of high perfusion voxels with relatively short mean bolus arrival times, which are likely to contain high fractions of gray matter. The additional condition on BOLD response was included to avoid introducing a bias by selection based on one contrast only. Setting a percentile rather than absolute z‐statistic threshold ensured ROIs of similar sizes across subjects and scans. This method also avoided the need to register to higher resolution structural images, which would invariably have introduced some smoothing and a reduction in spatial specificity. To emulate the more typical situation in which only one short‐TE ASL and one longer‐TE BOLD acquisition are acquired, the analysis was repeated with a separate gray matter ROI created for each BOLD TE.

Relative change in CBF was calculated on a voxelwise basis, assuming tagging efficiencies of 92% during normocapnia and 83% during hypercapnia 4. To remove excessively noisy data, voxels were excluded from ROI analysis if the CBF response to hypercapnia was negative or greater than 200%, or if the BOLD response was negative or greater than 15%. A regional M‐value was then calculated from mean CBF and BOLD responses within the remaining voxels (see Eq. [2]), using α = 0.18 (as measured by Chen and Pike 15 in a human MRI study investigating the venous response to hypercapnia) and β = 1.3 (appropriate for the field strength of 3T as per Bulte et al) 16.

## RESULTS

**Figure 1:**
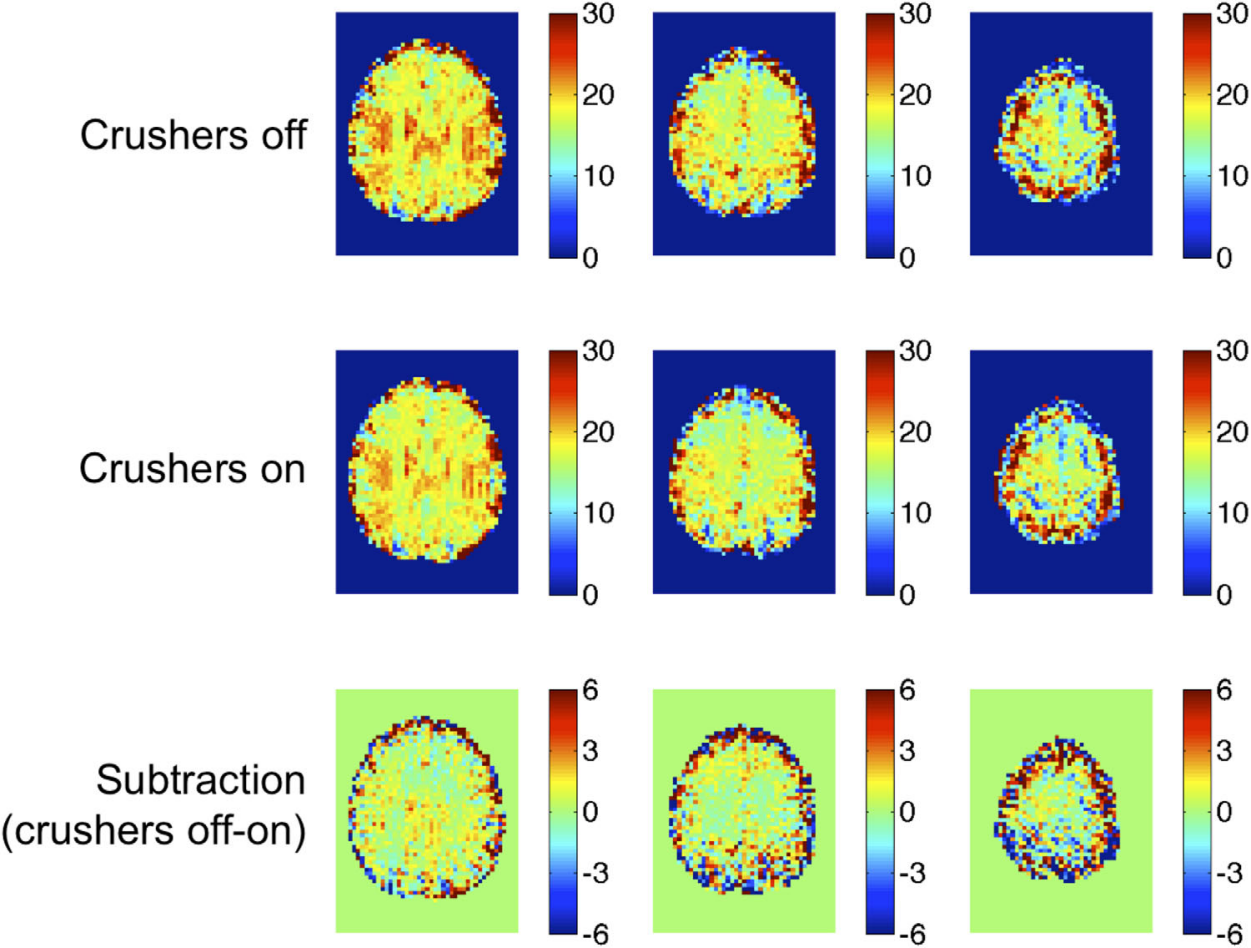
$R_{2}^{*}$ maps of a single representative subject.

All scans were carried out successfully by all eight volunteers. Mean CBF and BOLD changes to hypercapnia, with crushers on and off, are shown in Table 1. These were calculated within the gray matter ROI for each subject. R_2_* maps from one representative subject are shown in Figure 1. There was a small reduction in R_2_* when crushers were applied, consistent with a reduction in signal from the intravascular compartment.

**Table 1:** CBF and BOLD Responses to Hypercapnia^a^

	Crushers off	Crushers on
CBF/CBF_0_	1.39±0.05	1.45±0.09
% BOLD, 20ms	2.02±0.94	1.34±0.39
% BOLD, 35ms	2.83±1.23	2.30±0.61
% BOLD, 49ms	3.65±1.57	3.20±0.79
% BOLD, 64ms	4.33±1.69	3.94±0.97
% BOLD, 78ms	4.92±1.69	4.48±1.02

a Mean ± standard deviation results for CBF and BOLD changes during hypercapnia. CBF changes were only measured from the first echo at 20ms.

**Figure 2:**
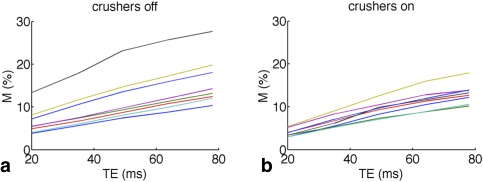
M as a function of echo time (TE), as typically measured in a calibrated fMRI experiment (**a**) and with intravascular crushers applied **(b**). Each line represents a different subject.

**Figure 3:**
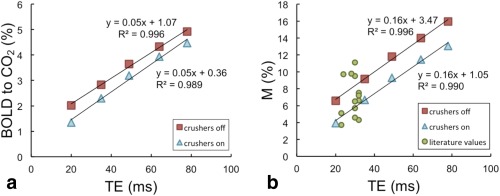
Mean BOLD response to hypercapnia (**a**) and mean M (**b**) from this study, with crushers turned on (blue triangles) and off (red squares). The results of linear fits have been added. Green circles show M results from past studies over a range of BOLD echo times (TE).

Figure 2 plots the mean gray matter M value as a function of TE for each of the eight subjects. Figure 3 shows the linear fitting to the group mean BOLD and M results as a function of echo time, for the cases where crusher gradients were turned on (blue triangles) and off (red squares). The linear model proved a good fit for the data, as demonstrated by R^2^ values of at least 0.98 in all cases.

**Figure 4:**
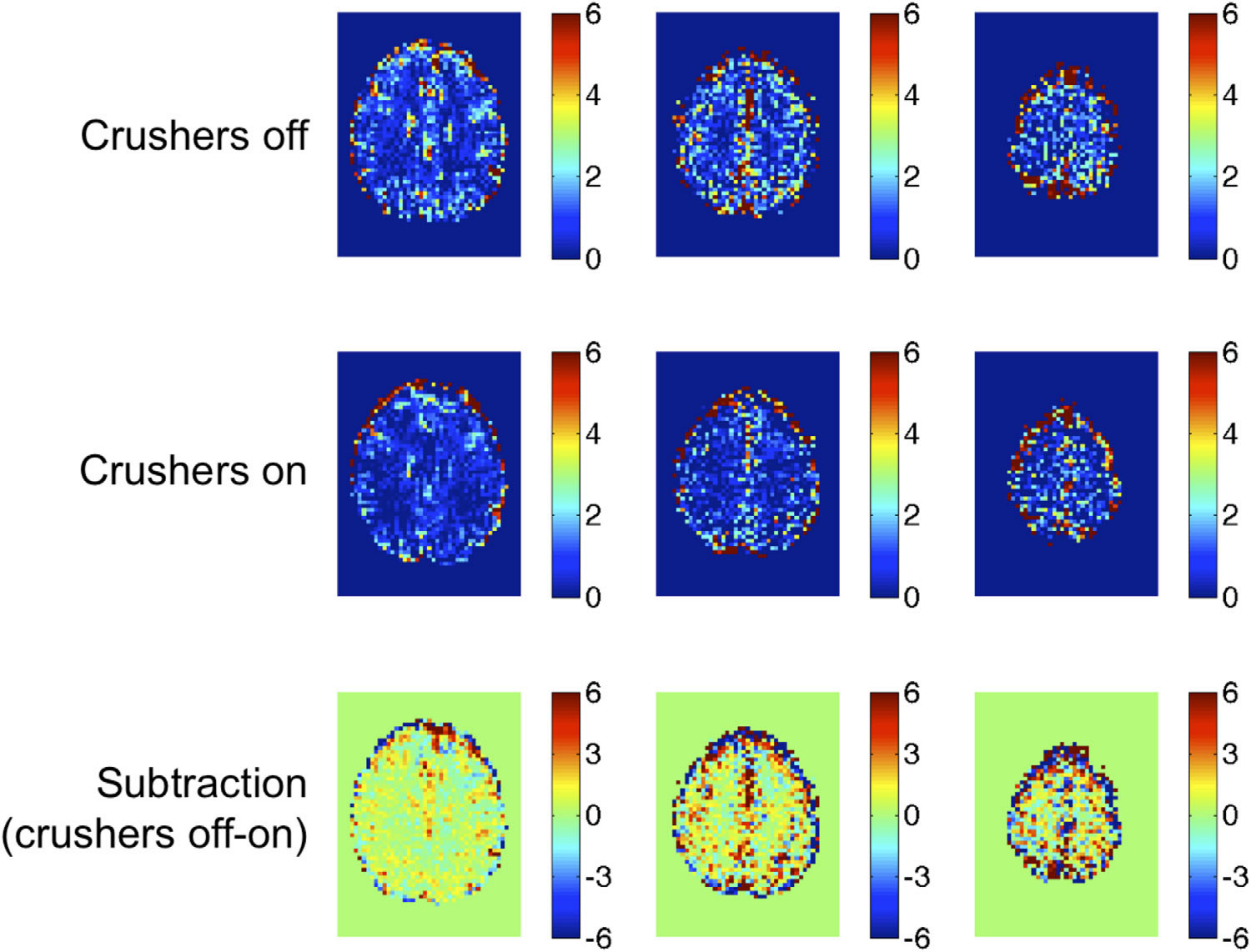
Percentage BOLD signal responses to hypercapnic stimuli in a single representative subject.

Figure 4 shows the percentage BOLD signal change (TE = 35 ms) in response to hypercapnia in a representative subject for the cases where crushing gradients were turned off and on. The difference image demonstrates that BOLD response is reduced throughout gray matter when crushers are turned on, presumably because these reduced the signal contribution from small veins throughout the brain. Table 1 and Figure 3a show the magnitude of this reduction in BOLD response within gray matter ROIs across all subjects. As a result, M values were consistently reduced when crushers were applied; for example, at the most typical BOLD echo time of 35ms, M was measured to be 9.2 ± 4.1% (mean ± standard deviation) without crushers, and 6.7 ± 1.4% with crusher gradients turned on. Y‐intercepts of 3.5% (crushers off) and 1.1% (crushers on) were observed, corresponding to the theoretical M at TE = 0, which is assumed by the model to be zero (see Eqs. [1] and [2]).

**Figure 5:**
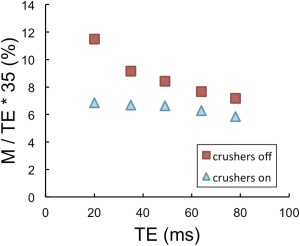
Mean M values measured with crushers on (blue triangles) and off (red squares), linearly scaled to an optimal echo time of 35 ms by assuming an intercept of zero.

Figure 5 shows the mean M values scaled to a TE of 35 ms, where the intercept of the linear fit has been assumed to be zero. If M were a direct linear function of TE, as predicted by Eqs. [1] and [2], Figure 5 would show that the normalized M was independent of TE. For the “crushers on” situation this is very nearly the case, with small deviations visible only at very long echo times (TE > 50 ms). However, when crushers are turned off, it is evident that the scaled M value decreases monotonically with TE, demonstrating that M is not a purely linear function of TE with an intercept of zero as has previously been assumed.

## DISCUSSION

The green circles in Figure 3b represent M values quoted in the literature, which have been taken from the following works: Ances et al 17, 18, Chen and Parrish 19, Chiarelli et al 3, 20, Gauthier et al 7, Leontiev and Buxton 21, Lin et al 22, and Perthen et al 23. M values found in this study fall well within this range.

As seen in Figure 3a, the BOLD signal change in response to a hypercapnic stimulus is not directly proportional to TE. The reduction in the intercept from 1.07 without crushers to 0.36 when crushers were applied suggests that the deviation from behavior predicted by Eq. [1] is likely due to intravascular signal contamination. The crusher gradients used in this sequence had a cutoff velocity of 1.9 cm/s and were only applied in the head‐foot direction; hence although they reduced the intravascular signal contribution, they will not have eliminated it completely.

The second assumption made by Eq. [1] is that the exponent is sufficiently small to allow for linearization of the exponential. For typical BOLD responses of ∼3% (at TE=35ms), the error introduced by this linearization in the estimation of $\Delta R_{2}^{*}$ is less than 2%. In individual voxels with larger BOLD responses—such as those containing large veins—this error will be increased. However, linearizing an exponential function and extrapolating this back to zero would tend to underestimate the value of an intercept, so this effect is unlikely to have contributed to the positive BOLD intercepts observed in this study.

Having seen that the BOLD response is not a directly linear function of TE, it follows that the same is true of M. Again, the y‐intercept (which represents the deviation from predicted behavior) was reduced but not eliminated when crushers were applied. In studies where two echoes are acquired (e.g., by using a dual‐echo or interleaved pulse sequence), it would be possible to estimate this intercept on a study‐ or subject‐specific basis, which may significantly improve the accuracy of scaling M to any desired echo time.

In Figure 5, the mean M values have been normalized to an echo time of 35ms, assuming a linear relationship with zero intercept. This gives an idea of the errors introduced by the commonly used linear scaling method. For the case where crushers are turned on (blue triangles), normalized M is almost a flat function of TE from 35 ms, meaning that any error in the scaling of M for typical BOLD echo times (30–50 ms) appears negligible. Even where crushers are turned off (indicated by red squares), the error introduced by the scaling is typically moderate. For example, scaling an experimental M with a BOLD TE of 49 ms to 35 ms yields an M of 8.4%, where the “true” 35 ms M value is 9.2%. However, scaling up from a shorter experimental echo time introduces a greater error, for example from 20 ms to 35 ms would give a scaled M of 11.5% compared with the actual value of 9.2%.

One must bear in mind that the scaling of M with TE is only relevant when comparing between studies that had different BOLD echo times. Most experiments that measure M do so to calculate changes in or absolute values of CMRO_2_; within a single study, the BOLD echo time chosen should not have any significant impact on these results. This is at the very heart of the calibrated MRI methodology: the parameter M by design varies with experimental conditions in such a way as to allow the conversion from BOLD signal changes to underlying changes in oxygen metabolism. However, as the current protocol did not include the performance of any neurological tasks, this data is not capable of testing the conjecture that measured CMRO_2_ is independent of BOLD echo time. As the theory behind the Davis model considers only extravascular BOLD signal contributions, we expect that the application of flow crushers would improve the accuracy of CMRO_2_ estimates, although at the expense of noisier ASL data because of a prolonged initial TE.

Echo time is not the only factor affecting M value; in addition to differences in physiology (resting blood volume, mean vessel size etc.), voxel size, field inhomogeneities, and scanner hardware will also affect its value. Thus one would expect a certain amount of variation in M values measured at different imaging centers, as is demonstrated by the substantial range in literature M values shown in Figure 3b. M values are generally scaled and quoted between studies for a coarse comparison only, and the additional error or variability introduced by assuming a linear scaling with zero intercept is likely to be small compared with these other considerations, even for the more common case where crusher gradients are not applied.

The choice of constraints used to define ROIs in functional MRI has been a controversial subject for many years. In the field of calibrated MRI, both ASL and BOLD signals are of interest, and ROIs have typically been created from voxels that are responsive to both contrasts in an attempt to avoid or minimize any biasing. In this study, significant BOLD responses to hypercapnia were required at 3 or more echo times, as well as good perfusion, in order to be labeled a “gray matter voxel”. This resulted in the creation of a common ROI for all echoes, ensuring that any observed trends are not mere artifacts of differences in ROIs (although it should be noted that excessively noisy voxels were individually removed from these ROIs during further analysis). To replicate the more common experimental situation in which only one BOLD‐weighted echo is acquired, the analysis was repeated with a separate gray matter ROI being created for each BOLD echo time. The trends observed were unchanged, however the average BOLD responses to hypercapnia were slightly increased (2.98 ± 1.35% with no crushers at 35 ms as compared to 2.02 ± 0.94% in main analysis), as were M values (9.5 ± 4.5% compared with 9.2 ± 4.1%). Continuing the example of a 20 ms ASL/35 ms BOLD acquisition with crusher gradients turned off, and defining a gray matter ROI based only on good resting ASL signal (see Methods section for a more detailed definition), an M value of 5.7 ± 2.3% was calculated. When using good BOLD response to hypercapnia at 35 ms TE as our only condition, this increases to 8.1 ± 2.8%, and by combining the two conditions we end up with 9.5 ± 4.5%. This demonstrates the importance of choosing and reporting ROI definitions carefully.

## CONCLUSIONS

M has been commonly assumed to vary in direct proportion with TE. We have shown that the relationship is indeed linear (R^2^ = 0.996 on data points averaged across eight subjects), but has a nonzero intercept. The value of the intercept is reduced when crusher gradients are added to the sequence, suggesting that the deviation from theory arises from the false assumption of negligible intravascular BOLD signal contribution. Where two echoes are acquired during a dual‐echo or interleaved sequence it would be possible to estimate the intercept on a study‐ or subject‐specific basis, which could significantly improve the accuracy of scaling M to a different echo time. However, in practice, the errors introduced by the assumption of a zero intercept are relatively small between typical BOLD echo times of 30–50 ms. In general we recommend against putting undue weight on agreements or disagreements in M values between different studies, especially when the echo times or ROIs used differ significantly, regardless of which scaling method is used.
